# Adaptive Point-Line Fusion: A Targetless LiDAR–Camera Calibration Method with Scheme Selection for Autonomous Driving

**DOI:** 10.3390/s24041127

**Published:** 2024-02-08

**Authors:** Yingtong Zhou, Tiansi Han, Qiong Nie, Yuxuan Zhu, Minghu Li, Ning Bian, Zhiheng Li

**Affiliations:** 1 Tsinghua Shenzhen International Graduate School, Tsinghua University, Shenzhen 518055, China; zhouyt21@mails.tsinghua.edu.cn; 2 Meituan, Lizedong Road, Chaoyang District, Beijing 100102, China; hantiansi@meituan.com (T.H.); nieqiong@meituan.com (Q.N.); 3 Department of Automation, Tsinghua University, Beijing 100084, China; yx-zhu21@mails.tsinghua.edu.cn; 4 Dongfeng Motor Group Co., Ltd., Wuhan 430058, China; liminghu@dfmc.com.cn (M.L.); biann@dfmc.com.cn (N.B.)

**Keywords:** LiDAR–camera extrinsic calibration, autonomous vehicles, targetless calibration, point-line fusion, deep learning, feature refinement, adaptive method selection, non-overlapping calibration

## Abstract

Accurate calibration between LiDAR and camera sensors is crucial for autonomous driving systems to perceive and understand the environment effectively. Typically, LiDAR–camera extrinsic calibration requires feature alignment and overlapping fields of view. Aligning features from different modalities can be challenging due to noise influence. Therefore, this paper proposes a targetless extrinsic calibration method for monocular cameras and LiDAR sensors that have a non-overlapping field of view. The proposed solution uses pose transformation to establish data association across different modalities. This conversion turns the calibration problem into an optimization problem within a visual SLAM system without requiring overlapping views. To improve performance, line features serve as constraints in visual SLAM. Accurate positions of line segments are obtained by utilizing an extended photometric error optimization method. Moreover, a strategy is proposed for selecting appropriate calibration methods from among several alternative optimization schemes. This adaptive calibration method selection strategy ensures robust calibration performance in urban autonomous driving scenarios with varying lighting and environmental textures while avoiding failures and excessive bias that may result from relying on a single approach.

## 1. Introduction

Autonomous vehicles are equipped with various sensors that perceive the environment, including LiDAR for depth information and cameras for color and texture data. By fusing these modalities [1], accurate semantic information can be constructed, which enables precise object segmentation and other functionalities. To achieve effective fusion of different sensor modalities, it is crucial to accurately calibrate the extrinsic parameters.

The target-based method is the most commonly used approach for LiDAR–camera calibration. This method uses markers such as chessboard patterns to establish data association and performs calibration by minimizing reprojection errors between different sensors [2,3,4]. This method often requires specific calibration setups and even manual intervention. Discontinuous LiDAR point clouds can cause incorrect data associations and calibration failure in the presence of noise.

Targetless calibration eliminates the need for artificial targets, making online calibration [5] more convenient. Researching calibration in natural scenes has become a popular area of interest in practical engineering. Some methods extract line and edge features [6,7] from LIDAR and cameras to establish a correlation between 3D point clouds and 2D points. Aligning edges directly can be a difficult task due to the different data modalities of the sensors. To address this issue, Castorena et al. [8] incorporated natural alignment of depth and intensity edges and used a Gaussian mixture model for parameter estimation. Mutual information-based methods are used to estimate the extrinsic parameters by maximizing the mutual information between the surface intensities measured by the sensor [9,10]. However, these methods have certain limitations, such as a high requirement for data quality and distribution, and a need for a significant overlap in the field of view between sensors.

Researchers have investigated various methods to tackle the problem of non-overlapping configuration. Ahmad et al. [11] proposed an approach that is based on the robot–world hand–eye calibration (RWHE) problem. Napier et al. [12] synthesized images from LiDAR reflectance values using a calibration between sensors and measured their alignment based on gradients. Taylor et al. [13] focused on estimating the motion of each sensor and performing extrinsic calibration based on the estimated motion relationship. Yao et al. [14] utilized the Normalized Information Distance (NID) metric to accurately estimate the extrinsic parameters between multiple cameras and a 2D LiDAR system. These methods may pose challenges in terms of computation and robustness.

In autonomous driving applications, an accurate pose of a LiDAR sensor is typically obtained by fusing multiple sensors such as GNSS, RTK, and LiDAR. To make the most of reliable data and make it easier to combine different types of data, we use a method called modality transformation. It transforms the challenge of aligning LiDAR and camera features into a matching and optimization problem on the image plane. This method allows for calibration in natural scenes even when no overlapping region exists between the sensors.

There have been attempts to explore methods for adaptive calibration. Liu et al. [15] proposed the use of an adaptive voxelization technique for calibrating small FoV LiDARs and cameras. The calibration has been formulated as a LiDAR Bundle Adjustment problem. Wen [16] proposed a method for adaptive calibration of LiDAR sensors used on roadsides. The method uses Kalman filtering and the RANSAC algorithm to align the LiDAR coordinate system with an ideal coordinate system. AFLI-Calib [17] uses adaptive frame length LiDAR odometry for self-calibration of LiDAR-IMU systems, enabling extrinsic calibration. Yao [18] proposed an adaptive joint calibration method for camera and 3D LiDAR systems, which uses a single planar calibration board. The method optimizes the extrinsic parameters of the camera along with the rotation and translation between the LiDAR and camera coordinates. However, there is no adaptive method to select the suitable scheme for targetless calibration without overlapping. Therefore, we propose an adaptive selection strategy to achieve good calibration performance under varying lighting and image texture conditions. After transforming the calibration problem into an optimization problem within a visual SLAM system, we consider introducing line features to enhance the robustness of the system by providing additional constraints. Targetless calibration is just a minor component of autonomous driving systems. Due to limited GPU training resources, we employ pre-trained models and supplementary algorithms to reduce resource consumption and achieve a coarse-to-fine outcome.

## 2. Related Work

Line features are commonly used in computer vision tasks such as image registration, 3D reconstruction, and object detection. The Hough Transform (HT) [19] is a classical technique that converts the image space into a parameter space, enabling the extraction of line segments through peak detection. Optimization approaches such as the Probabilistic Hough Transform (PPHT) [20] were proposed to improve its performance. Algorithms like LSD [21,22], Linelet [23], MCMLSD [24], and others group pixels in local image regions based on their gradient directions and fit them into line segments. However, these algorithms often result in higher false positive rates. DeepLSD [25], a deep learning-based algorithm, can automatically extract line segments but requires a large amount of accurately labeled training data. EDLine [26] uses gradient magnitude to fit edge line segments but is prone to fragmentation. In addition, having too many line features can cause notable misalignment and bias in optimization due to inaccuracies. To tackle problems like inaccurate line extraction and incorrect positive identifications, AG3line [27] employs the geometric constraints of lines to extract line segments, achieving state-of-the-art performance in line feature extraction. This method is used to detect line features by extracting environmental texture lines and reducing false positives and fragmentation. We use this method to detect line features.

Point-Line Visual Odometry, a variant of Visual Odometry (VO), utilizes point and line features for accurate real-time motion estimation. Struct-SLAM [28] uses building structure lines for localization and map construction, while PL-SLAM [29] improves localization accuracy with line features. VP-SLAM [30] optimizes rotation and refines translation using line feature vanishing points. However, these methods are limited to small indoor scenes. To enhance adaptability, deep learning-based approaches like AIRVO [31] extract robust features in challenging lighting conditions. Deep learning methods [32] often require abundant labeled data for network training and may lack the flexibility to adjust the feature extraction process. To overcome these challenges, we propose a novel method that combines deep learning features and eliminates the need for additional training data. Our approach allows for fine-tuning of line segment feature results while applying visual odometry for extrinsic calibration.

Line feature matching is crucial in visual SLAM (Simultaneous Localization and Mapping). LBD [33] is a widely used method for line feature matching. It uses binary descriptors to represent line segments, encoding local gradient information along the line segment. The similarity between line segment features is then measured using Hamming distance, which has rotational and scale invariance. The line feature matching algorithm based on deep learning also has impressive results. Some combined semantics and Hough Transform for line feature detection in natural scenes [34], some [35] introduce a novel algorithm that combines the position and appearance information of points and lines through a graph neural network to construct enhanced line feature descriptors, thereby improving matching capabilities, and some [36] propose a new end-to-end wireframe evaluation metric to obtain continuous line segments. However, these methods typically require substantial computational resources. Lim et al. [37] proposed a method that utilizes optical flow to obtain predicted lines and perform fusion. This method enables fast and effective matching and removal of duplicates, resulting in significant computational savings. We employ this method for line feature tracking and achieved promising results.

The feature point method utilizes a set of specific feature points, such as ORB [38], SURF [39], or SIFT [40], to represent the environment. In texture-rich environments, this approach improves localization accuracy by extracting accurate feature points. However, in environments with weak textures, this method may fail to produce reliable results. Direct methods minimize photometric error for feature tracking and pose estimation. The direct method has good robustness to changes in illumination and dynamic environments, and can maintain good performance in low-texture or repetitive texture environments. In order to combine the advantages of two methods, we adopt two feature extraction matching methods in parallel and choose them adaptively.

The photometric error is used to evaluate the intensity difference between local images. Direct photometric optimization has been effectively utilized in the fields of optical flow [41] and visual odometry [42]. In recent years, some extensible methods have emerged. EDPLVO [43] introduced photometric error into the constraints of line features, improving the accuracy of point-line visual odometry. PixSfM [44] combines the high-dimensional features of deep learning and performs generalized optimization on the positions of 2D pixel points. Our work applies this method to adjust the positions of line segment features.

We present a new method for LiDAR–camera calibration, without requiring a target in autonomous driving scenarios. Our contributions are as follows:

- We introduce a novel method for calibrating LiDAR and camera systems without requiring overlapping fields of view. Once we obtain a highly accurate LiDAR pose, we can use it to assist with calibration effectively. This practical approach aligns with the needs of real-world autonomous driving applications.
- We extend the high-dimensional error used in SFM point feature optimization to the line feature. Our method utilizes the accurate feature extraction abilities of deep learning-based techniques and refines line feature position without the need for retraining.
- We present an adaptive strategy for selecting a higher-precision calibration method that remains robust and performs well under varying urban lighting and image texture conditions.

This paper is organized as follows: Section 3 describes the Transformation and Optimization methods. Section 4 outlines the experimental conditions. The results are shown in Section 5 and Section 6 concludes the research and discusses future applications.

## 3. Materials and Methods

We will provide an overview of the entire method framework, followed by a detailed explanation of the algorithms used in each component.

### 3.1. The Overview of System

There are two modules in this method: (i) Transformation and (ii) Optimization. The Transformation module determines the camera pose by aligning and interpolating the LiDAR pose and external parameters. This helps in correlating data between different modalities. The obtained camera pose is then fed into the Optimization section to perform the triangulation process.

The Optimization module is divided into two sections: pre-optimization and iterative optimization. In the pre-optimization section, the results of a single optimization and the information from line feature filtering are combined to adaptively select the appropriate optimization method. The final calibration results are achieved through iterative optimization methods.

**Figure 1:**
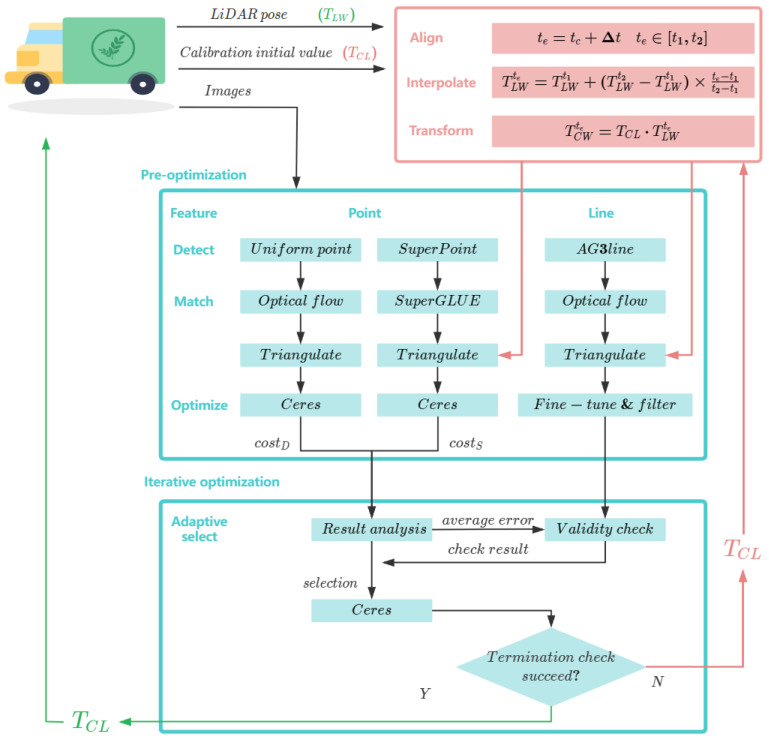
The overview of the proposed method. In the proposed method, we begin with the pre-optimization stage, where we use two parallel methods for extracting point features and matching them between frames. At the same time, we use the initial pose values provided by the Transformation module to triangulate the point features. Based on the results of point feature triangulation and line feature selection, we determine the appropriate point feature optimization scheme and decide whether to include line features.

Figure 1 shows the details of this method.

### 3.2. Transformation

It is commonly observed that conventional techniques of calibrating sensors usually involve projecting 3D point clouds onto 2D images for feature identification. However, researchers often encounter significant errors with this method due to factors like noise. To overcome this issue, we suggest establishing data correlation based on pose rather than features. For instance, if we have two consecutive LiDAR timestamps, $t_{1}$ and $t_{2}$, the average camera exposure time as Δt, the camera timestamp as $t_{c}$, and the image frame timestamp as $t_{e}$, we can utilize these values to determine the correlation between the data:

(1) $$t_{e}=t_{c}+\Delta t,\;t_{e}\in \left[t_{1},\;t_{2}\right]$$

In order to determine the LiDAR pose at the time of image capture, interpolation is utilized. Specifically, linear interpolation is used for translation, while Euler angles or quaternion interpolation can be used for rotation. Our method entails constructing the initial pose of the image frame through the precise LiDAR pose and initial values of external parameters with perturbations to simulate real-world scenarios:

(2) $$T_{CW}^{t_{e}}=T_{CL}\cdot T_{LW}^{t_{e}}$$

**Figure 2:**
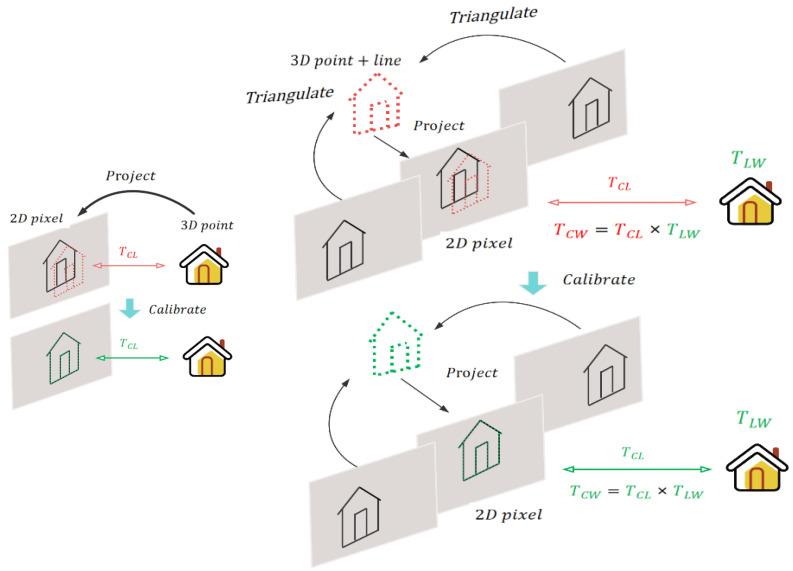
Typical sensor calibration methods involve projecting 3D point clouds onto 2D images to identify features. However, our proposed method establishes data correlation based on pose instead of features. This involves triangulating point and line features on the image plane using pose information and adjusting feature positions and external parameters to minimize reprojection error. Then, we achieve calibrated external parameters.

In the process demonstrated in Figure 2, we input $T_{CW}$ to triangulate point and line features on an image plane. After this, the system adjusts the positions of these features and external parameters by differentiating the reprojection error. The aim is to minimize the overall reprojection error and obtain calibrated external parameters.

### 3.3. Pre-Optimization

#### 3.3.1. Feature Extraction and Triangulation

Firstly, we extract point and line features from the image and match them between frames. After obtaining the poses from the transformation module, we triangulate the features to determine their 3D positions. After that, we optimize the rotation vector, 3D point features, and line features by constructing the reprojection error.

**Figure 3:**
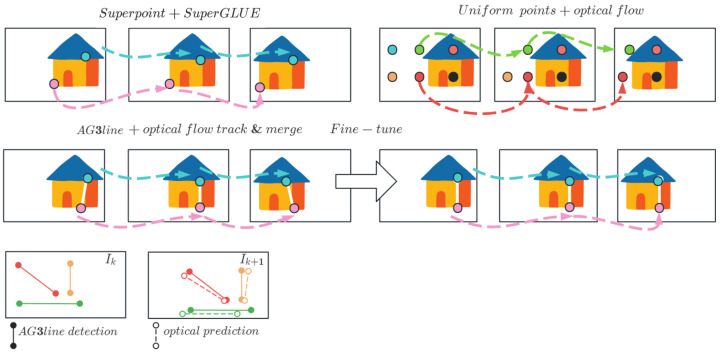
We employ two parallel methods for processing point and line features. For point feature processing, we use the SuperPoint algorithm in conjunction with SuperGlue, uniform sampling of points, and optical flow. To extract line features, we make use of the AG3line method and fine-tune it using an expanded photometric error approach. Tracking of the same feature point between frames is shown using arrows and same color point features.

Both feature point methods are used for extraction and matching, with the most suitable method dynamically selected. In the fast method, features are uniformly positioned throughout the image, similar to object detection using VDO-SLAM [45]. This ensures that feature information is available even in environments with less distinct textures. We use optical flow for inter-frame feature tracking. If the number of inlier tracks falls below a certain level (which is set at 1200 by default), new features are detected and added to the map. The feature point method extracts up to 1200 SuperPoint [46] feature points per frame and uses the SuperGLUE [47] model for matching. The AG3line method is used to extract line features, as explained in [27], while prediction and fusion are achieved using the optical flow method proposed in [37]. The entire process is illustrated in Figure 3. It is important to note that this process uses only existing deep learning models and methods, and does not require additional data for model training.

AG3line is a feature extraction method that works well. However, the method that uses gradients to extract lines can sometimes be inaccurate and have a negative impact on optimization. Changes in lighting and viewing angles can lead to edge detection inaccuracies when extracting line features.

The Pixel-perfect SFM method (PixSFM) [44] was developed to tackle the problem of inaccurate extraction of 2D feature points in 3D reconstruction tasks. PixSFM uses high-dimensional features in classification and introduces a new error function for all tracks associated with the same point. This allows for the adjustment of feature point positions before triangulation. We have also extended this optimization approach to refine the positioning of line features.

PixSFM assigns a confidence weight *w* to each pair of 2D feature points associated with feature point *j*, considering all tracks M(j). The measurement error of a feature point can be defined as follows:

(3) $$E^{j}=\sum_{\left(u,v)\in M(j\right)}w_{uv}\left|\right|I_{i}\left[p_{u}\right]-I_{k}\left[p_{v}\right]{\left|\right|}_{\gamma }$$

In this context, the variable *I* represents an image, while *p* denotes the position of a feature point within the image. Once the endpoints of line features have been extracted, the Brief [48] descriptor is computed for each endpoint. This computation helps to determine the confidence between consecutive pairs of points. It is important to remember that line features may occasionally have breaks and occlusions, causing a lack of correspondence between endpoints. However, this method enables the endpoints to adjust towards the direction closer to the gradient edge, without requiring precise endpoint matching.

#### 3.3.2. Adaptive Selection Strategy

We optimize external parameters by using the reprojection error of point features obtained from both the fast and feature point methods. The cost reduction ratio for the fast method and the feature point method are denoted as $p_{F}^{e}$ and $p_{S}^{e}$, respectively. The retention rates of feature points are denoted as $p_{F}^{r}$ and $p_{S}^{r}$. In the same method, when there is an increase in the error in external parameters, the cost reduction ratio also increases. To reflect the decrease in accuracy of the same optimization method as the error in external parameter increases, we introduce the optimization confidence $c_{F}$ and $c_{S}$:

(4) $$c_{i}=e^{h(p^{e},p^{r},\alpha ,\beta )}$$

(5) $$h\left(p^{e},p^{r},\;\alpha ,\beta \right)=\alpha \frac{-p^{e}}{f_{i}\left(p^{r}\right)}$$

(6) $$f_{i}\left(p\right)=p^{\beta_{i}},i\in \left\{F,S\right\}$$

where f(·),α,β represent scaling function and scaling coefficients. When external parameters have the same initial values, we choose the method with a smaller h(·) because it results in a faster error decrease after mapping, which is considered to have a higher level of optimization accuracy.

We now move on to perform a line validity check. We consider all tracks M(j) associated with line *j*. The triangulation results obtained from different observations are denoted as $L(P_{u_{1}},P_{u_{2}})$ and $L(P_{v_{1}},P_{v_{2}})$, where $P\in R^{3}$ and u,v∈M(j). In a sequence, *d*, *v*, and γ, respectively, represent the length range, variance, and threshold of line feature triangulation results. If the following conditions are met, we can conclude that incorporating line features into optimization will not result in significant oscillations:

(7) $$\left|\right|P_{u_{i}}-P_{v_{i}}{\left|\right|}_{2}<\gamma_{L},\;i=1,2$$

(8) $$d<\gamma_{d}$$

(9) $$v<\gamma_{v}$$

If the initial perturbation is a small angle of 0.6°, we can move forward with integrating line features for optimization. To check if the initial perturbation is small enough, we assess the average error of point features during the pre-optimization process. If the error meets the required condition, we can proceed with the optimization of line features:

(10) $$e_{k}<\delta_{k},\;k\in \left\{F,S\right\}$$

### 3.4. Iterative Optimization

After analyzing the previous module, we determined the optimization approach for both point and line features. The overall cost function is as follows:

(11) $$min\sum_{i\in I}\left(\sum_{j\in P(i)}e_{ij}^{P}+\theta \sum_{k\in L(i)}e_{ik}^{L}\right),\;\theta =0,1$$

Images, points, and line segments are denoted by I, P, and *L*, respectively.

(12) $$e^{P}=x-\pi_{P}\left(\xi ,X\right)$$

(13) $$e^{L}=\eta d\left(\xi ,L\right)$$

The function π is used to project a 3D point *X* from the world coordinate system onto a 2D image plane. The external parameters to be optimized are represented by ξ∈se(3). We use the result of the previous optimization as the initial value for the external parameters and then continuously iterate until the number of iterations reaches the threshold. We use weighted values, represented by η, to adjust the influence of line feature length and track count. By doing so, the line features’ residual error is reduced. We give greater importance to line features that are almost perpendicular to the horizontal plane, as they provide more precise triangulation. We sorted vertical and horizontal lines based on their angles relative to the horizontal plane post-triangulation. We included them in the optimization in the following proportion: frames–vertical–horizontal = 600:90:10.

### 3.5. Line Feature Representation

Plücker coordinates can represent 3D line segments with 6-DoF using normal vector **n** and direction vector **d**:

(14) $$L{\left(n^{T},d^{T}\right)}^{T}\in R^{6}$$

QR decomposition [49] can produce a non-redundant representation for line segment features since they have only four degrees of freedom:

(15) $$\left[n\;d\right]=\left[\frac{n}{\left||n|\right|}\;\frac{d}{\left||d|\right|}\;\frac{n\times d}{\left||n\times d|\right|}\right]\left[\begin{array}{cc} \left||n|\right| & 0\\ 0 & \left||d|\right|\\ 0 & 0 \end{array}\right]$$

The normal vector **n** and direction vector **d** of a line feature can be obtained by triangulating the two endpoints of the line segment:

(16) $$L=\left[\begin{array}{c} X_{1}\times X_{2}\\ w_{1}X_{2}-w_{2}X_{1} \end{array}\right]=\left[\begin{array}{c} n\\ d \end{array}\right]$$

where the homogeneous coordinates of 3D endpoints are denoted as $X={\left[x,y,z,w\right]}^{T}$. When the observing planes are represented as $\Pi ={\left[\pi_{1},\pi_{2},\pi_{3},\pi_{4}\right]}^{T}$, the Plücker matrix can also be expressed as:

(17) $$L^{*}=\left[\begin{array}{cc} d_{\times } & n\\ -n^{T} & 0 \end{array}\right]=\Pi_{0}\Pi_{1}^{T}-\Pi_{1}\Pi_{0}^{T}$$

### 3.6. Measurement Model

To obtain the representation of the projected line $l_{c}$ on the 2D image plane, the Plücker coordinates $L_{w}$ in the world coordinate system are first transformed into $L_{c}$ in the camera coordinate system. This transformation is followed by utilization of the internal parameter projection matrix $K_{l}$:

(18) $$L_{c}=\left[\begin{array}{cc} R_{cw} & {\left[t_{cw}\right]}_{\times }R_{cw}\\ 0 & R_{cw} \end{array}\right]L_{w}$$

(19) $$l_{c}=\left[\begin{array}{c} l_{1}\\ l_{2}\\ l_{3} \end{array}\right]=K_{l}n_{c}=\left[\begin{array}{ccc} f_{y} & 0 & 0\\ 0 & f_{x} & 0\\ -f_{y}c_{x} & -f_{x}c_{y} & f_{x}f_{y} \end{array}\right]n_{c}$$

The residual is calculated as the distance between the endpoints $x_{s},x_{e}$ of the observed line and the projected line:

(20) $$e^{L}=d\left(x_{s},x_{e},l_{c}\right)={\left[\begin{array}{c} \frac{x_{s}^{T}l_{c}}{\sqrt{l_{1}^{2}+l_{2}^{2}}},\frac{x_{s}^{T}l_{c}}{\sqrt{l_{1}^{2}+l_{2}^{2}}} \end{array}\right]}^{T}$$

It is important to note that a single line feature can have an infinite number of feature points. We propose adding residuals at the midpoint of the line segment $x_{m}$ to improve optimization compared to the two endpoints:

(21) $$e^{L}=d\left(x_{s},x_{m},x_{e},l_{c}\right)={\left[\begin{array}{c} \frac{x_{s}^{T}l_{c}}{\sqrt{l_{1}^{2}+l_{2}^{2}}},\frac{x_{m}^{T}l_{c}}{\sqrt{l_{1}^{2}+l_{2}^{2}}},\frac{x_{s}^{T}l_{c}}{\sqrt{l_{1}^{2}+l_{2}^{2}}} \end{array}\right]}^{T}$$

## 4. Experimental Conditions

### 4.1. Dataset and Device

**Figure 4:**
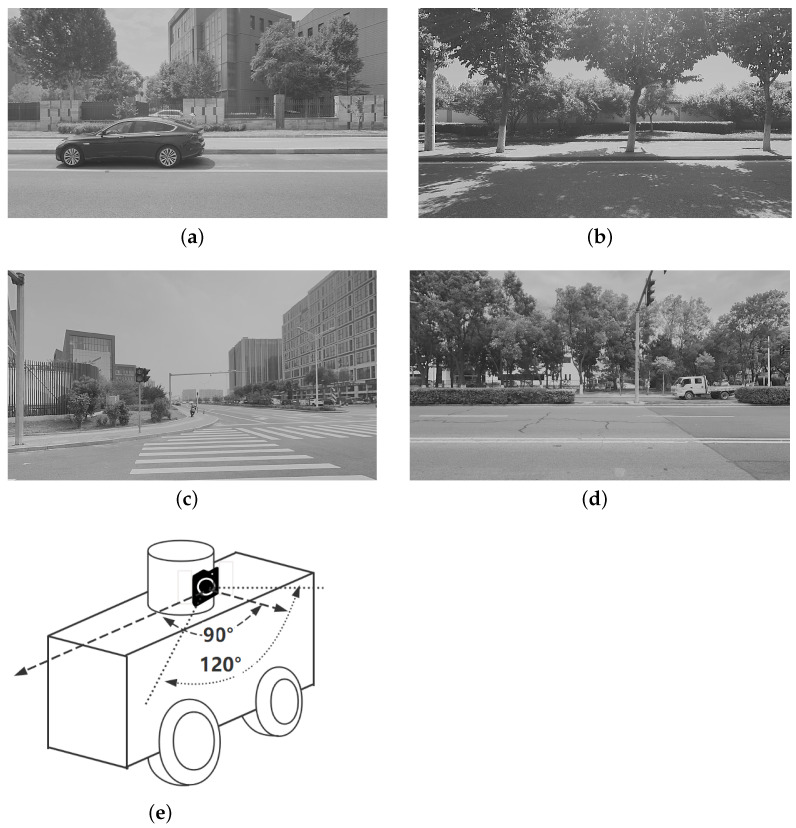
Our dataset includes various urban scenarios with different lighting conditions, environmental textures, and distances. (**a**,**c**): Urban scenarios with less shadow and occlusion or rich environmental textures. (**b**,**d**): Urban scenarios with severe shadow and occlusion or unclear environmental features. (**e**): For our study, we used a side camera, which is fixed above the vehicle’s body. The camera is positioned at 90-degree angle from the center of the vehicle’s body towards its front.

We captured six sets of urban street scenes using monocular side-facing cameras mounted on autonomous delivery vehicles, as shown in Figure 4e. These datasets show different textures, lighting, and distance, which can be seen in Figure 4. Two datasets were well-lit with rich textures (Figure 4a,c), while two others were shaded by trees with weak textures (Figure 4b,d). The remaining two had mixed lighting and textures. Autonomous vehicles encounter varying lighting and shadow conditions in some frames of their datasets, while other frames show consistent scene characteristics.

Extracting accurate line features in outdoor urban scenes is more difficult compared to indoors due to the impact of distance and environmental content. To ensure that we had enough and precise line features, we decided to extract features from a consecutive range of 450 to 600 frames in each sequence. The time interval between two adjacent frames is roughly 0.02 s. If line features were not necessary, we could reduce the number of datasets. Simultaneously, we adjusted actual devices with deviations in their external parameters.

The external parameters set by the factory during the manufacturing process are known as the initial values. Obtaining an accurate LiDAR pose requires a fusion algorithm that combines GNSS, RTK, GPS, and LiDAR, which is beyond the scope of our work. For our experiments, we used a monocular fisheye camera with a 120-degree field of view (FOV). Our experimental environment was equipped with a single NVIDIA GeForce GPU, and no additional GPU resources were required for training. We relied on this GPU to perform all of our experiments. We also demonstrated the calibration effect by projecting 3D points into a 2D plane.

### 4.2. Evaluation Metric

We converted the extrinsic rotation that requires calibration into Euler angles. To optimize the process, we applied perturbation angles of 0.0°, 0.15°, 0.25°, 0.4°, 0.6°, 0.8°, and 1.0° in the roll, pitch, and yaw directions, which are commonly used convergence values. This allowed us to compare the absolute errors of the four correction methods to the ground truth after a single optimization.

## 5. Results

### 5.1. Fine-Tuning of Line Segments

**Figure 5:**
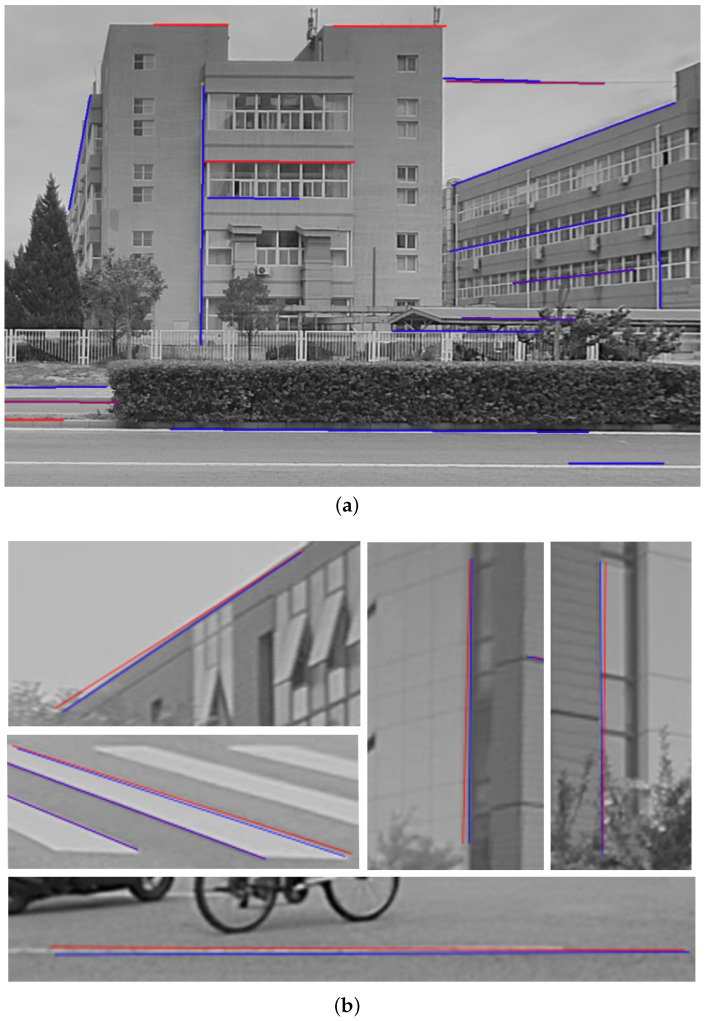
The figures above show the results obtained after extracting and fine-tuning line features. (**a**) demonstrates that AG3line can effectively extract scene line features that are clear, coherent, and with very few stray lines. In (**b**), the results of line feature optimization using the extended photometric error are displayed. The red lines represent the original lines with deviations, while the blue lines represent their positions after fine-tuning. The optimal position is situated closer to the edge.

After fine-tuning, we were able to achieve highly accurate line features. In Figure 5, the red line segments represent the edge segments that were initially imprecise. On the other hand, the blue line segments depict the optimized and accurate ones, which helped reduce errors caused by inaccurate feature extraction.

### 5.2. Pre-Optimization

**Figure 6:**
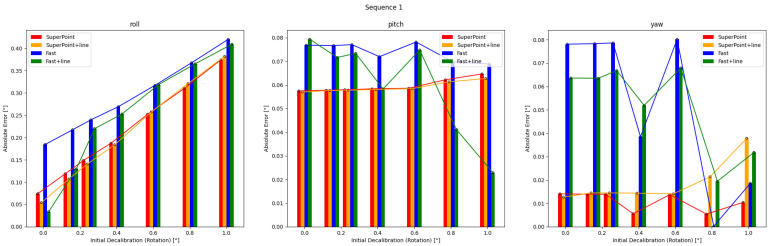
Results obtained from the four calibration methods under different extrinsic parameter errors for Sequence 1 are presented. The feature point method has a significant advantage in scenes with sufficient illumination and clear textures.

**Figure 7:**
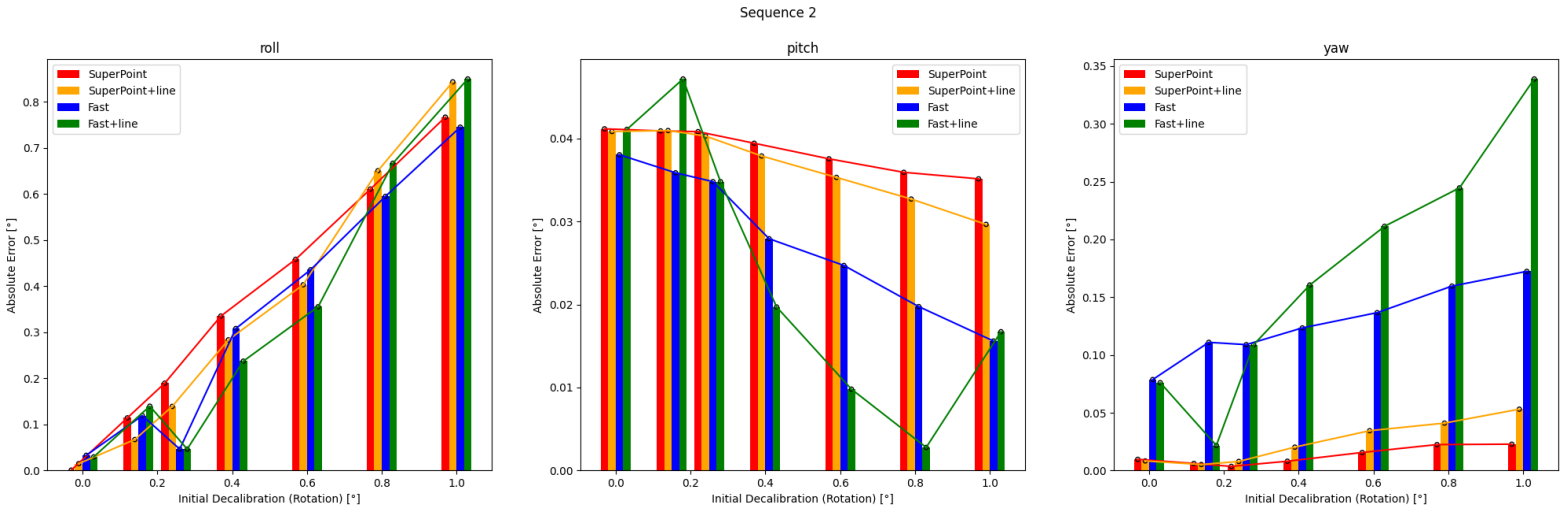
Results obtained from the four calibration methods for Sequence 2 are presented under different extrinsic parameter errors. The direct method outperforms the other methods in scenes with weak illumination and unclear textures.

**Figure 8:**
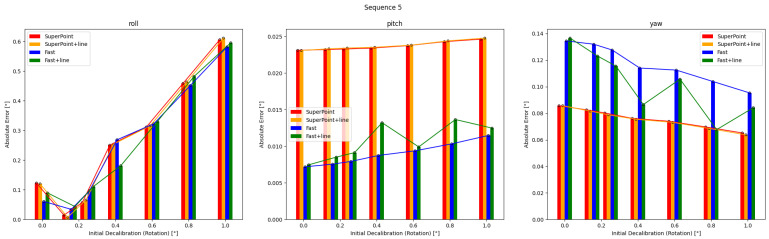
Results obtained from four calibration methods are presented for Sequence 5 under varying extrinsic parameter errors. Calibration result may change with initial values in more complex mixed scenes.

The results of single optimizations for three sequences with varying initial perturbations are displayed in Figure 6, Figure 7 and Figure 8. The details are in Table A1, Table A2, Table A3, Table A4, Table A5 and Table A6. It can be seen that the roll correction exhibits more significant deviations as compared to the pitch and yaw directions. However, the latter are usually corrected within an error range of 0.15°. The pitch error is always less than 0.1°.

In Sequence 1, the SuperPoint-based method shows a noticeable advantage. In Sequence 2, the direct approach demonstrates a clear advantage. These scenes are shown in Figure 4b. In Sequence 3, the effectiveness of different correction methods fluctuates to some extent.

The optimization method that incorporates line features has positive effects at smaller perturbation angles (≤0.6°). As the initial disturbances become more severe, the line features may gradually have negative effects. In scenes with sufficient lighting and clear environmental textures, the feature point method has a distinct advantage. However, in low-texture or dimly-lit environments, the direct method proves more robust. Additionally, as the initial error increases, the gap between the optimization effects of the two methods gradually decreases.

### 5.3. Adaptive Selection Method

**Figure 9:**
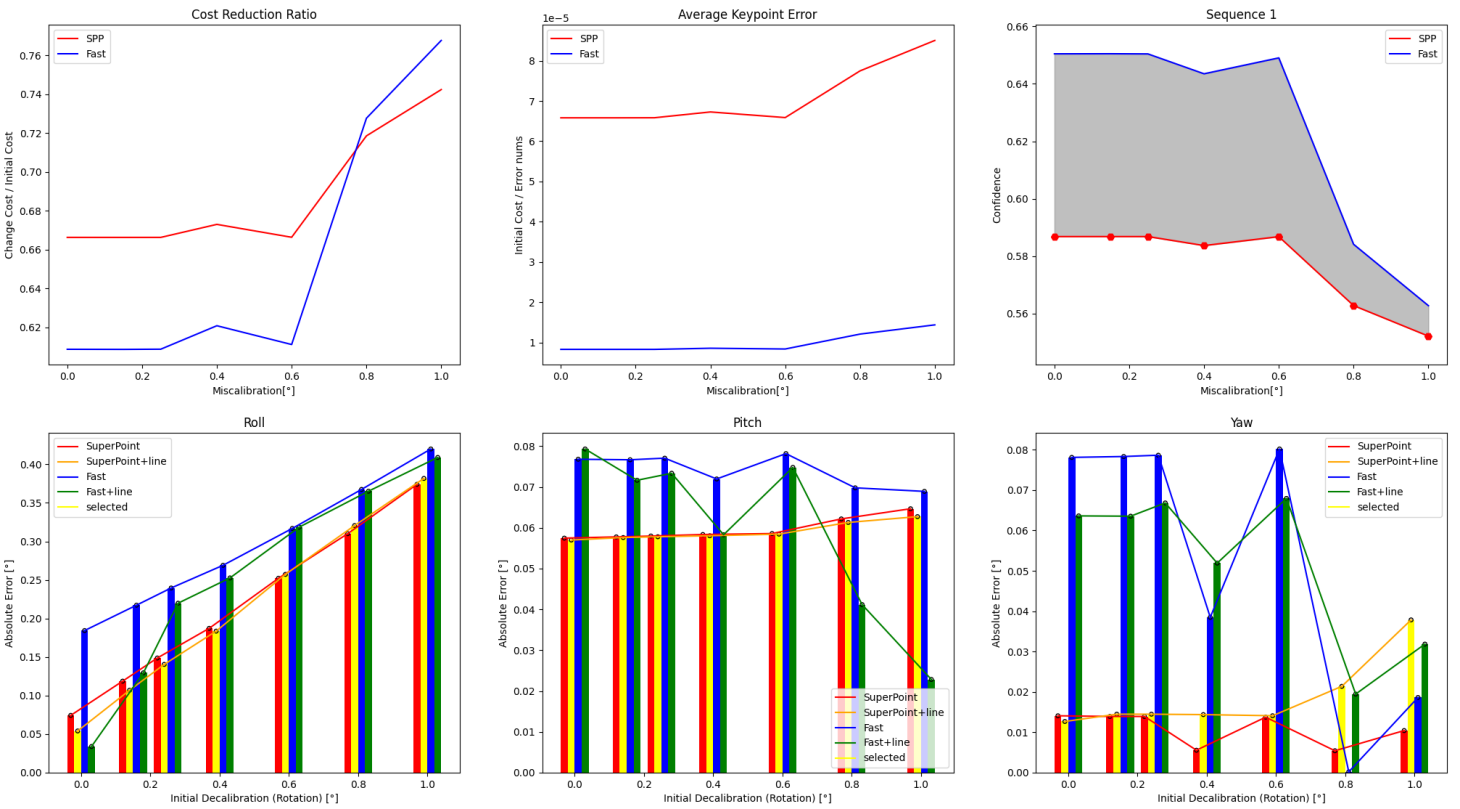
In Sequence 1, during the pre-optimization process, the confidence curves of the two feature methods displayed a clear separation, indicating a significant difference in their effects. Our adaptive selection method chose the SuperPoint-based feature method and combined it with line features for optimization under varying initial values.

**Figure 10:**
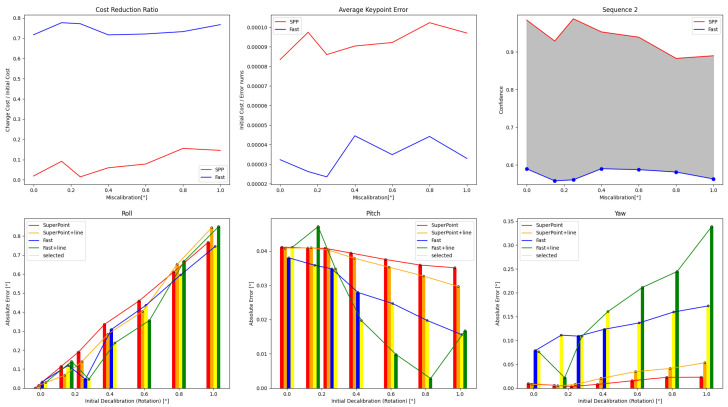
During the pre-optimization process for Sequence 2, the confidence curves of two feature methods were analyzed and showed a clear distinction between them, indicating a significant difference in their effects. Our adaptive selection method opted for the direct method combined with line features for optimization under small initial perturbations.

**Figure 11:**
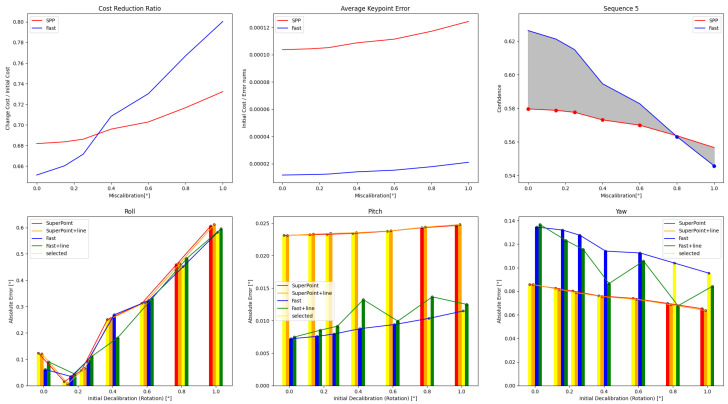
For Sequence 5, different schemes can be selected under different initial values as the confidence reading curve has crossed.

**Figure 12:**
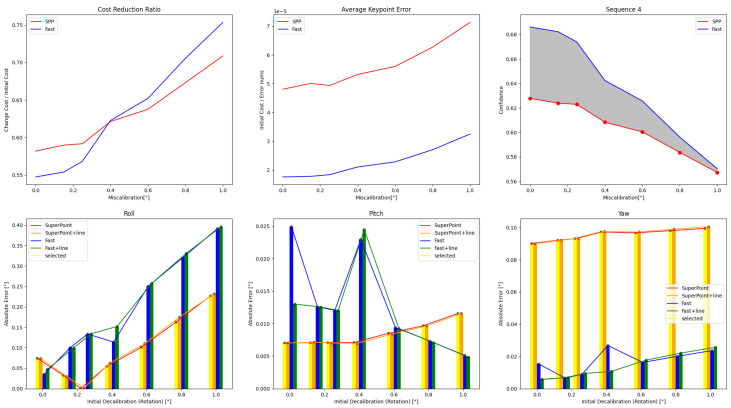
The line features in Sequence 4 are of poor quality and have a high variance in length, which exceeds the threshold. Due to this, the adaptive strategy did not select these line features for optimization. Instead, a better solution was chosen to correct the roll.

**Figure A1:**
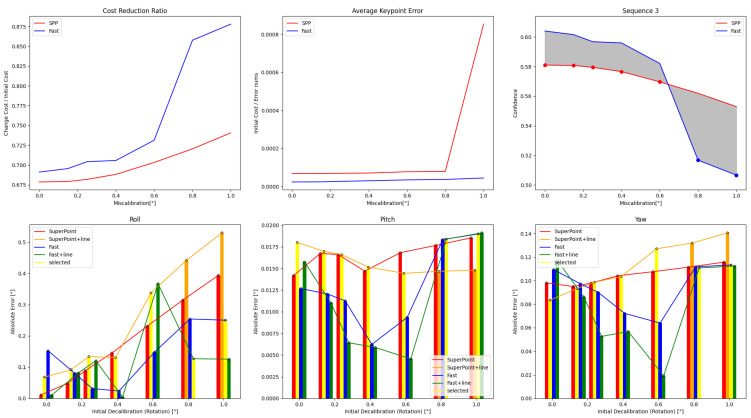
In Sequence 3, different schemes can be selected under different initial values as the confidence reading curve has crossed. As the error increases, our selection method first chooses the optimization method that combines SuperPoint-based feature method and line features. Then, it chooses the fast method.

**Figure A2:**
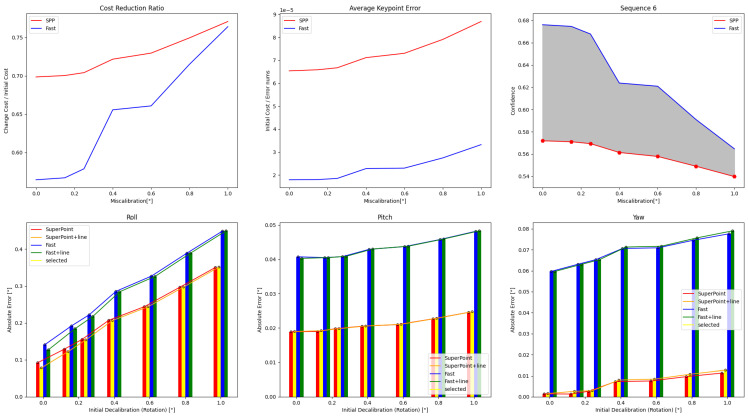
In Sequence 6, during the pre-optimization process, the confidence curves of the two feature methods displayed a clear separation, indicating a significant difference in their effects. Our adaptive selection method chose the SuperPoint-based feature method and combined it with line features for optimization under varying initial values.

Different methods for correcting roll, pitch, and yaw may have varying effects, requiring a trade-off. Typically, the roll direction with the highest deviation is prioritized. Figure 9, Figure 10, Figure 11 and Figure 12 demonstrate the selection methods for the four aforementioned sequences. The rest are in Figure A1 and Figure A2. As the initial perturbations increase, the ratio of cost reduction in the pre-optimization process and the average error of feature points both increase, leading to a reduction in the confidence level determined by our criteria. We use the cost reduction ratio and the average error of the feature points in pre-optimization to decide whether to include line features in the iterative optimization process. A positive hexagonal marker on the confidence curve shows that the point feature optimization method has been selected.

The main objective is to calibrate the roll, as errors in pitch and yaw calibration are less significant. It is clear that in more than half of the cases, the chosen methods are generally good options and rarely result in the selection of the worst-case method. This helps avoid significant deviations caused by a single optimization method.

### 5.4. Iterative Optimization

It is important to note that the use of pre-trained models may lead to errors without retraining. As depicted in Figure 9, Figure 10, Figure 11, Figure 12, Figure A1 and Figure A2, when the initial external parameter is accurate, i.e., the initial disturbance is 0°, the external parameter obtained by using the pre-trained model for feature extraction, matching, and single optimization shows a deviation below 0.12° near the roll angle, a deviation below 0.08° near the pitch angle, and a deviation below 0.14° near the yaw angle. Thus, iterative optimization is necessary, taking the last calibration result as the initial value, to achieve a better outcome after several iterations of calibration.

**Figure 13:**
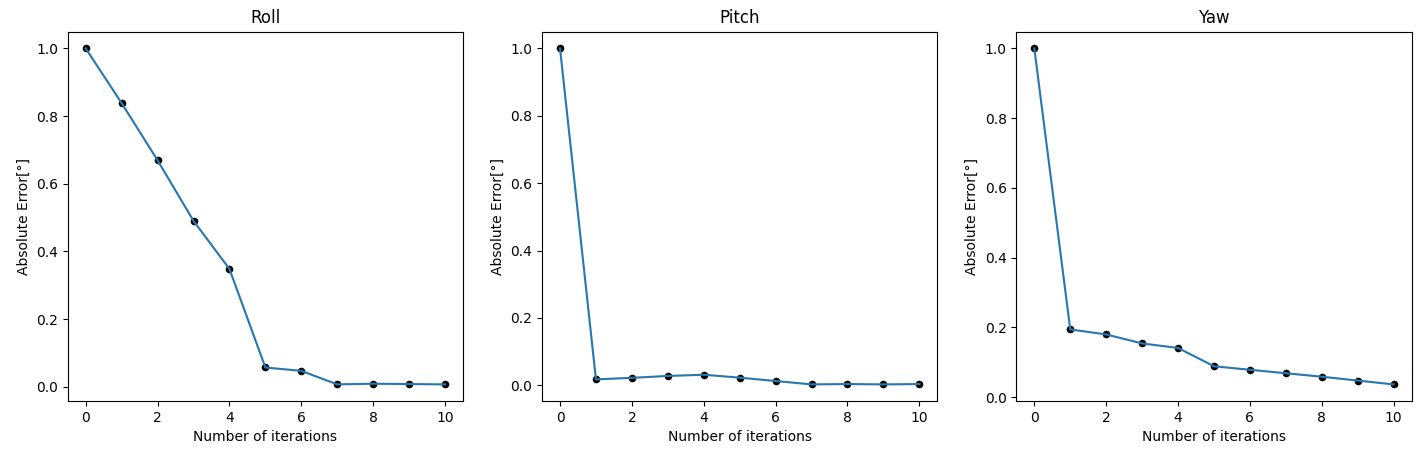
The calibration effect of iterative optimization on Sequence 2.

The performance of Sequence 2 within the iteration optimization module is illustrated in Figure 13. In each iteration, the previously converged extrinsic calibration result is used as the initial value for optimization. With each iteration, the calibration results gradually converge.

### 5.5. Visualization of the Projection

**Figure 14:**
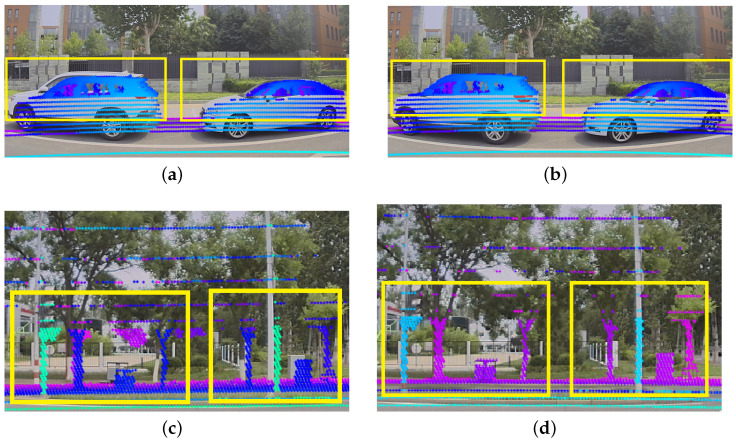
Example of calibration results. In (**a**,**c**), the initial calibration values have an error of 1.0° in the roll, pitch, and yaw directions. After applying our calibration method, (**b**,**d**) can achieve a level close to the true values. The projection accuracy of objects such as car edges, poles, and trees has been enhanced.

**Figure 15:**
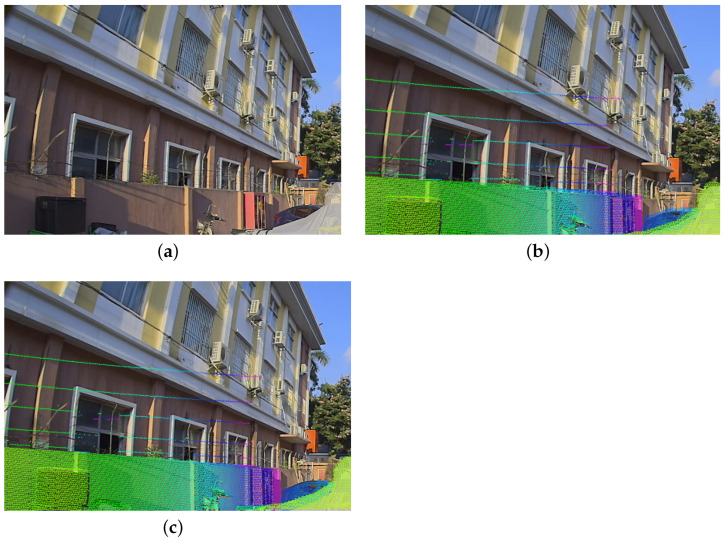
Example of calibration results. To better visualize the results, we project the LiDAR point cloud onto the image plane, as shown in the figure above. (**a**) shows the original image. In (**b**), the device’s initial external parameters have a deviation of around 0.3° in the roll and 0.6° in the pitch. This results in noticeable projection deviations in walls and objects. In (**c**), after calibration through our iterative method, the error has been significantly reduced to less than 0.1°. Consequently, the projection is now essentially correct.

The visualization of the projection effect is shown in Figure 14 and Figure 15. Figure 14 represents the simulation effect of adding rotation near the true value and we add 1 degree of disturbance based on the true value of the external parameters. Figure 15 shows the calibration experiment for a device with actual deviation. The external parameters of the current device have a deviation, and calibration is used to obtain accurate values.

### 5.6. Time Consumption

To illustrate the resource consumption, we compiled the average time consumption for feature extraction, matching, and optimization on a single frame as shown in Table 1. The great advantage of the direct method is its short time consumption. The method based on deep learning takes more time. Therefore, under different resource conditions, the best plan can be selected. In situations that require real-time processing, the fast method, or a combination of the fast method and line characteristics, can be used to achieve a negligible increase in processing time ranging from $1\times 10^{-8}$ s to $1\times 10^{-7}$ s. Dealing with situations that require a moderate level of real-time performance, it is possible to fine-tune the line segment features. When real-time processing is not needed, an adaptive strategy scheme can be utilized. The SPP method and the SPP method combined with fine-tuning of line features are the most time-consuming methods, adding 0.055 s to 0.11 s to the processing time per frame.

## 6. Discussion and Conclusions

In autonomous driving engineering, GNSS, RTK, and LiDAR are often used as multiple sensors to obtain precise vehicle positions. We aim to use this information for automatic calibration of vehicles in natural scenes, avoiding the impact of feature alignment noise. To do this, we establish a connection between the LiDAR and camera through the vehicle’s poses. By doing so, we convert the external parameter calibration problem into an optimization problem based on visual data. The end goal is to improve the vehicle calibration accuracy by combining the strengths of LiDAR and visual images.

According to the research results, the feature-based calibration method showed better performance in well-lit and textured scenes. In contrast, the method based on uniformly sampled feature points performed better in scenes with weak lighting and textures. In mixed scenes, the effectiveness of the calibration method depended on the specific situation. When the initial error was below 0.6°, incorporating line feature constraints with point features reduced calibration error. However, optimizing the parameters of the line features became more difficult as the initial error increased, so adding line feature constraints might have a negative impact.

To make the most of the texture information in an image, we want to incorporate the constraints of line features. However, experiments have demonstrated that the importance of line feature constraints in outdoor scenes is not as significant as in many indoor scenes. Firstly, the uncontrollability of line features is higher, and their length and distribution cannot be artificially restricted or manipulated. Secondly, the farther the distance, the greater the deviation caused by the triangulation of line features may be. When the camera is placed too far from the object, a wide rectangular shape in the image may appear as small as a single pixel. Thirdly, it is possible that outdoor shadows could affect line feature extraction. Fourthly, in natural environments devoid of man-made structures, reliable line features are difficult to extract. Moreover, the residual magnitude of point features is typically around $1\times 10^{-8}$ pixels, while the residual magnitude of line features can go up to 2 pixels. The discrepancy in residual values suggests that line features, particularly inaccurate ones, will have a significant impact on the optimization process. Therefore, it is essential to not only accurately extract line features but also limit their weight and screen them when added to the optimization. We can further improve the accuracy of line features by using the expanded photometric error method through fine-tuning. However, fine-tuning is a time-consuming process and can only be adopted when there are no time and resource constraints.

We observed that finding a single optimization solution that works well for all cases is challenging due to the complexity of real-life scenarios. Additionally, it is not easy to automatically adjust parameters. Therefore, we propose having multiple methods as alternatives. We compare the optimization results under the same external conditions and select the scheme with the best results, allowing for adaptive strategy selection in different scenarios. Our approach can choose either the best or second-best optimization strategy in more than half of the cases and rarely selects the worst outcome. This adaptive approach can prevent any single solution failure in specific scenarios.

Overall, our method provides a simplified and efficient way for calibrating LiDAR–camera systems. We have developed a targetless calibration method for LiDAR–camera calibration that eliminates the need for artificial targets, making online calibration possible. Common techniques for the calibration of LiDAR data involve aligning edges and using information-based algorithms. Unlike these approaches, our method does not require the noise of the LiDAR point cloud data in the calibration process to be handled, nor does it require consideration of the error of 3D-2D feature alignment. Moreover, our approach does not necessitate the overlap of the field of view among various sensors. Our approach does not start with the original features, unlike typical academic solutions. Instead, we begin by utilizing accurate LiDAR poses to transform the challenge of aligning 3D and 2D data into an optimization problem of pose associations from an engineering point of view, which utilizes the existing information to its fullest potential. To overcome the limitations of adapting a single scheme to all scenes, we propose an adaptive strategy that achieves good calibration performance across different lighting and image textures. We have noticed that the current calibration method for the LiDAR–camera combined line feature is only suitable for indoor scene calibration. To address this issue, we have introduced line segment features for outdoor automatic driving scenes. This will improve overall robustness. Additionally, we have identified potential problems that may arise when using line segment features in outdoor scenes. Our approach is different from the calibration method that relies on deep learning. We utilize a pre-trained model to extract and match features. Then, we complement it with the expanded photometric error method to fine-tune the position of line features. This approach allowed us to achieve better matching performance, reduce the consumption of training resources, and attain the calibration effect from coarse to fine without the need to retrain the actual scene.

Please be aware that we utilize the pre-trained models developed by the original creators of SuperPoint and SuperGLUE, without training them on real datasets. This approach helps to reduce resource consumption. However, if the training model undergoes any changes, the parameters of the mapping confidence curve may require adjustment to suit the new situation. It is important to note that our method may not be suitable for scenes that contain a large number of moving objects as they can adversely affect the optimization of line features. Moreover, it is crucial to verify the accuracy of the LiDAR trajectory before utilizing this method by checking for any noticeable deviation in the projection process. We hope that in the future, more adaptive solution options will be available to ensure that the system is robust in various complex real-world scenarios.

## Figures and Tables

**Table 1:** The time consumption of various calibration methods is as follows: (**a**) SPP represents SuperPoint feature point extraction and SuperGLUE feature matching method. (**b**) Fast represents the uniform sampling points extraction and optical flow matching method. (**c**) Line represents the AG3line extraction, optical flow matching, and fine-tuning method.

Method	Detect [s]	Match [s]	Fine-Tuning [s]
SPP	0.0115	0.0424	-
Fast	$4.4985\times 10^{-9}$	$5.5606\times 10^{-8}$	-
Line	$9.1940\times 10^{-8}$	$1.3932\times 10^{-8}$	0.0507

## Data Availability

The data are not publicly available due to privacy.

## Appendix A

We provide additional information for the adaptive selection method curve of the two remaining sequences.

We provide a table of error values for six sequences after a single calibration.

**Table A1:** Sequence 1.

Method	Roll/Pitch/Yaw Absolute Error [°]	Absolute Miscalibration [°]						
		**0.0000**	**0.1500**	**0.2500**	**0.4000**	**0.6000**	**0.8000**	**1.0000**
SPP		0.0743	0.1188	0.1487	0.1873	**0.2522**	**0.3101**	**0.3741**
SPP+line		0.0541	**0.1070**	**0.1405**	**0.1836**	0.2573	0.3206	0.3818
Fast		0.1843	0.2173	0.2396	0.2691	0.3169	0.3675	0.4199
Fast+line		**0.0339**	0.1296	0.2198	0.2527	0.3186	0.3652	0.4090
SPP		0.0575	0.0578	0.058	0.0583	0.0586	0.0622	0.0647
SPP+line		**0.0570**	**0.0576**	**0.0578**	**0.0580**	**0.0584**	0.0613	0.0627
Fast		0.0768	0.0767	0.0771	0.0720	0.0782	0.0698	0.0689
Fast+line		0.0793	0.0716	0.0734	0.0583	0.0748	**0.0412**	**0.0228**
SPP		0.0141	**0.0139**	**0.0139**	**0.0056**	**0.0137**	0.0054	**0.0104**
SPP+line		**0.0127**	0.0145	0.0145	0.0144	0.0141	0.0214	0.0379
Fast		0.0781	0.0783	0.0787	0.0385	0.0802	**0.0002**	0.0186
Fast+line		0.0636	0.0635	0.0668	0.0519	0.0679	0.0195	0.0319

**Table A2:** Sequence 2.

Method	Roll/Pitch/Yaw Absolute Error [°]	Absolute Miscalibration [°]						
		**0.0000**	**0.1500**	**0.2500**	**0.4000**	**0.6000**	**0.8000**	**1.0000**
SPP		**0.0012**	0.1287	0.2134	0.3772	0.5157	0.6871	0.8627
SPP+line		0.0170	**0.0758**	0.1567	0.3191	0.4535	0.7319	0.9487
Fast		0.0373	0.1341	**0.0524**	0.3468	0.4902	**0.6693**	**0.8382**
Fast+line		0.0335	0.1572	**0.0524**	**0.2671**	**0.3999**	0.7501	0.9558
SPP		0.0463	0.0460	0.0459	0.0443	0.0422	0.0404	0.0395
SPP+line		0.0459	0.0461	0.0453	0.0426	0.0397	0.0368	0.0333
Fast		**0.0428**	**0.0403**	**0.0391**	0.0314	0.0278	0.0222	**0.0175**
Fast+line		0.0462	0.0530	**0.0391**	**0.0222**	**0.0110**	**0.0031**	0.0188
SPP		0.0110	0.007	0.0041	**0.0091**	0.0176	**0.0253**	**0.0257**
SPP+line		**0.0094**	**0.0059**	**0.0089**	0.0228	**0.0388**	0.0462	0.0598
Fast		0.0886	0.1248	0.1226	0.1389	0.1537	0.1796	0.1939
Fast+line		0.0856	0.0245	0.1226	0.1806	0.2378	0.2751	0.3812

**Table A3:** Sequence 3.

Method	Roll/Pitch/Yaw Absolute Error [°]	Absolute Miscalibration [°]						
		**0.0000**	**0.1500**	**0.2500**	**0.4000**	**0.6000**	**0.8000**	**1.0000**
SPP		0.0112	**0.0486**	0.0887	0.1450	0.2313	0.3146	0.3935
SPP+line		0.0683	0.0918	0.1332	0.1303	0.3374	0.4416	0.5297
Fast		0.1517	0.0803	**0.0312**	0.0238	**0.1475**	0.2539	0.2500
Fast+line		**0.0102**	0.0815	0.1197	**0.0045**	0.3667	**0.1265**	**0.1251**
SPP		0.0142	0.0167	0.0166	0.0147	0.0168	0.0177	0.0185
SPP+line		0.0180	0.0170	0.0166	0.0152	0.0144	**0.0147**	**0.0148**
Fast		**0.0127**	0.0120	0.0113	0.0062	0.0094	0.0183	0.0190
Fast+line		0.0158	**0.0110**	**0.0064**	**0.0059**	**0.0045**	0.0184	0.0191
SPP		0.0981	0.0950	0.0979	0.1041	0.1076	0.1115	0.1158
SPP+line		**0.0835**	0.0932	0.0986	0.1035	0.1271	0.1320	0.1407
Fast		0.1094	0.0974	0.0901	0.0722	0.0639	0.1118	0.1134
Fast+line		0.1173	**0.0861**	**0.0526**	**0.0567**	**0.0191**	**0.1108**	**0.1124**

**Table A4:** Sequence 4.

Method	Roll/Pitch/Yaw Absolute Error [°]	Absolute Miscalibration [°]						
		**0.0000**	**0.1500**	**0.2500**	**0.4000**	**0.6000**	**0.8000**	**1.0000**
SPP		0.0862	0.0378	**0.0011**	**0.0632**	**0.1174**	**0.1894**	**0.2642**
SPP+line		0.0853	**0.0343**	0.0024	0.0728	0.1282	0.2035	0.2696
Fast		**0.0410**	0.1166	0.1552	0.1322	0.2912	0.3728	0.4555
Fast+line		0.0554	0.1158	0.1558	0.1768	0.2996	0.3851	0.4604
SPP		**0.0082**	**0.0082**	**0.0082**	0.0082	0.0099	0.0112	0.0134
SPP+line		**0.0082**	0.0083	**0.0082**	**0.0081**	**0.0098**	0.0112	0.0135
Fast		0.0290	0.0146	0.0140	0.0267	0.0109	0.0085	0.0059
Fast+line		0.0151	0.0146	0.0140	0.0284	0.0107	**0.0082**	**0.0056**
SPP		0.1047	0.1070	0.1082	0.1130	0.1123	0.1140	0.1156
SPP+line		0.1046	0.1070	0.1084	0.1133	0.1131	0.1151	0.1168
Fast		0.0178	**0.0077**	**0.0104**	0.0311	**0.0189**	**0.0233**	**0.0274**
Fast+line		**0.0068**	0.0079	0.0109	**0.0124**	0.0206	0.0256	0.0298

**Table A5:** Sequence 5.

Method	Roll/Pitch/Yaw Absolute Error [°]	Absolute Miscalibration [°]						
		**0.0000**	**0.1500**	**0.2500**	**0.4000**	**0.6000**	**0.8000**	**1.0000**
SPP		0.1537	0.0186	**0.0712**	0.3131	**0.3899**	0.5736	0.7575
SPP+line		0.1500	**0.0093**	0.0819	0.3174	0.3945	0.5794	0.7642
Fast		**0.0761**	0.0427	0.1222	0.3360	0.4031	**0.5647**	**0.7275**
Fast+line		0.1122	0.0547	0.1386	**0.2265**	0.4132	0.6028	0.7444
SPP		0.0289	0.0290	0.0291	0.0293	0.0297	0.0304	0.0308
SPP+line		0.0289	0.0291	0.0293	0.0294	0.0298	0.0305	0.0310
Fast		**0.0090**	**0.0095**	**0.0099**	**0.0109**	**0.0117**	**0.0129**	**0.0144**
Fast+line		0.0093	0.0106	0.0114	0.0165	0.0124	0.0171	0.0156
SPP		**0.1071**	0.1032	0.1002	0.0951	0.0924	0.0871	0.0815
SPP+line		**0.1071**	**0.1020**	**0.0985**	**0.0943**	**0.0914**	0.0856	**0.0796**
Fast		0.1681	0.1649	0.1595	0.1425	0.1406	0.1298	0.1191
Fast+line		0.1707	0.1538	0.1444	0.1082	0.1321	**0.0846**	0.1052

**Table A6:** Sequence 6.

Method	Roll/Pitch/Yaw Absolute Error [°]	Absolute Miscalibration [°]						
		**0.0000**	**0.1500**	**0.2500**	**0.4000**	**0.6000**	**0.8000**	**1.0000**
SPP		0.1083	0.1515	0.1826	0.2434	0.2867	**0.3488**	**0.4118**
SPP+line		**0.0918**	**0.1433**	**0.1809**	**0.2424**	**0.2863**	0.3492	0.4129
Fast		0.1657	0.2255	0.2611	0.3355	0.3841	0.4574	0.5277
Fast+line		0.1494	0.2166	0.2556	0.3339	0.3831	0.4573	0.5284
SPP		**0.0222**	**0.0223**	**0.0233**	**0.0241**	**0.0247**	**0.0266**	**0.0288**
SPP+line		0.0223	0.0226	**0.0233**	0.0242	0.0248	0.0268	0.0291
Fast		0.0479	0.0476	0.0479	0.0504	0.0514	0.0538	0.0566
Fast+line		0.0473	0.0476	0.048	0.0505	0.0515	0.054	0.0568
SPP		**0.0017**	**0.0016**	**0.0031**	**0.0085**	**0.0089**	**0.0113**	**0.0133**
SPP+line		0.0018	0.0031	0.0034	0.0094	0.0099	0.0126	0.0149
Fast		0.0702	0.0742	0.0767	0.0830	0.0833	0.0876	0.0912
Fast+line		0.0701	0.0741	0.0767	0.0838	0.0843	0.0889	0.0929
